# Cigarette Smoking and Risk of Breast Cancer in a New Zealand Multi-Ethnic Case-Control Study

**DOI:** 10.1371/journal.pone.0063132

**Published:** 2013-04-30

**Authors:** Fiona McKenzie, Lis Ellison-Loschmann, Mona Jeffreys, Ridvan Firestone, Neil Pearce, Isabelle Romieu

**Affiliations:** 1 International Agency for Research on Cancer, Lyon, France; 2 Centre for Public Health Research, Massey University, Wellington, New Zealand; 3 School of Social and Community Medicine, University of Bristol, Bristol, United Kingdom; 4 London School of Hygiene and Tropical Medicine, London, United Kingdom; The University of Auckland, New Zealand

## Abstract

**Background:**

The association between breast cancer and tobacco smoke is currently unclear. The aim of this study was to assess the effect of smoking behaviours on the risk of breast cancer among three ethnic groups of New Zealand women.

**Methods:**

A population-based case-control study was conducted including breast cancer cases registered on the New Zealand Cancer Registry between 2005 and 2007. Controls were matched by ethnicity and 5-year age-group. Logistic regression was used to estimate the association between breast cancer and smoking at different time points across the lifecourse, for each ethnic group. Estimated odds ratios (OR) were adjusted for established risk factors.

**Results:**

The study comprised 1,799 cases (302 Māori, 70 Pacific, 1,427 non-Māori/non-Pacific) and 2,540 controls (746 Māori, 191 Pacific, 1,603 non-Māori/non-Pacific). There was no clear association between smoking and breast cancer for non-Māori/non-Pacific women, although non-Māori/non-Pacific ex-smokers had statistically significant increased risk of breast cancer when smoking duration was 20 years or more, and this remained significant in the fully adjusted model (OR 1.31, 95% CI 1.03 to 1.66). Māori showed more consistent increased risk of breast cancer with increasing duration among current smokers (<20 years OR 1.61, 95% CI 0.55 to 4.74; 20+ years OR 2.03, 95% CI 1.29 to 3.22). There was a clear pattern of shorter duration since smoking cessation being associated with increased likelihood of breast cancer, and this was apparent for all ethnic groups.

**Conclusion:**

There was no clear pattern for cigarette smoking and breast cancer incidence in non-Māori/non-Pacific women, but increased risks were observed for Māori and Pacific women. These findings suggest that lowering the prevalence of smoking, especially among Māori and Pacific women, could be important for reducing breast cancer incidence.

## Introduction

Breast cancer is the most commonly registered cancer among New Zealand women; it accounts for 28% of all new female registrations and 16% of female cancer deaths [1]. Ethnic disparities have been observed in the distribution of the disease, with New Zealand's indigenous Māori women experiencing 40% higher registration rates and 43% higher mortality rates than non-Māori women [1]. Indigenous Māori comprise approximately 15% of the total New Zealand population. People originating from the United Kingdom and Europe make up approximately 77% of the population while the remaining major ethnic groupings comprise those from Asian countries (approximately 10%) and from the Pacific Islands (approximately 7%) [2].

Tobacco smoke is the most important known cause of cancer and has been associated with an extensive list of specific cancers [3], [4], [5]. There are a number of studies on the association between tobacco smoke and breast cancer risk [6], [7], [8], [9], [10], [11], [12], [13], [14], [15]; the possibilities of smoking being protective due to anti-estrogenic effects of tobacco [16], [17], [18], and being a risk factor due to the carcinogens in tobacco have both been posited [19], [20]. However, to date, findings from these studies continue to be controversial, and there remains no clear association, either positive or negative, with breast cancer risk. In 2009, the International Agency for Research on Cancer decided that there was limited evidence of a positive association between active smoking and breast cancer risk [21]. In the same year, the Canadian Expert Panel on Tobacco Smoke and Breast Cancer Risk concluded that there was an association between smoking and breast cancer that was consistent with causality [22].

Furthermore, there is also some evidence to suggest that exposure to tobacco smoke at particular time points [6], such as prior to first birth, may be associated with elevated breast cancer risk as undifferentiated breast cells are more sensitive to the carcinogen constituents of tobacco [23], [24]. The aim of this study was therefore to assess the effects of smoking at different points across the lifecourse on the risk of breast cancer in three ethnic groups of New Zealand women: Māori, Pacific, and non-Maori/non-Pacific women.

## Methods

### Ethics statement

Consent was obtained from all study participants and ethical approval was granted by the Central Health and Disability Ethics Committee (WGT/03/12/126) of New Zealand.

### Data availability

Ethics approval was granted for collection of information explicitly for the purposes of this study and the data are not publicly available.

### Study population

The New Zealand Breast Cancer Study, a population-based case-control study, was conducted to investigate risk factors throughout the lifecourse for breast cancer among three different ethnic groupings in New Zealand: Māori, Pacific, and non-Māori/non-Pacific women. The study design and methods have been published previously, and therefore will only be described briefly here [25]. All women with a primary invasive breast cancer registered on the New Zealand Cancer Registry (NZCR) between 1^st^ April 2005 and 30^th^ April 2006 were eligible for inclusion. To ensure sufficient numbers of cases, the eligible time period was extended for a further year to 30^th^ April 2007 for Māori and Pacific women. Control women were recruited from the New Zealand electoral roll, on which registration is mandatory for all New Zealand residents from 18 years of age. Controls were matched on ethnicity and frequency matched on 5-year age bands. The response rate among cases was 78% in non-Māori/non-Pacific women, 46% in Pacific, and 81% in Māori; for controls response was 57% in non-Māori/non-Pacific women, 15% in Pacific, and 38% in Māori.

### Data collection and smoking assessment

All participants completed comprehensive questionnaires on lifetime behaviours including socio-demographics; lifestyle; and reproductive and medical histories. Information on smoking was based on questions regarding current smoking and previous smoking patterns, including average amount per day at age 20 and 40 years. Participants were asked if they had ever smoked, now or in the past; at what age they started smoking regularly; and whether they are a current smoker. Current and former smokers were asked to select from pre-specified categories, on average, how many cigarettes they smoked in a day: under 10; 10 to 19; 20 or more; or whether they did not smoke at that particular age (for ages 20 and 40 years). Smoking duration was derived from deducting the age of starting to smoke regularly (initiation) from the age of stopping for ex-smokers, and for current smokers, the age at interview for controls, and age at diagnosis for cases. For ex-smokers the number of years since quitting was derived from the difference between age at interview (age at diagnosis for cases) and the age at which they ceased smoking. Duration prior to first birth was estimated by subtracting age of smoking initiation from the age of first live birth.

### Covariates

Body mass index (BMI) was estimated from participant self-reported information (weight in kilograms divided by height in metres squared). Information on exercise was based on questions about the average frequency of leisure activities over the preceding year (Godin Leisure Time Exercise Questionnaire) [26], [27]. Alcohol was based on the frequency of consumption during the preceding year. Other covariates included were age, age at menarche, history of maternal breast cancer, oral contraceptive (OC) ever use, HRT ever use, parity/number of live births, and socioeconomic position (SEP). The New Zealand Deprivation Index 2006 [28] was used as a measure of SEP. The Deprivation Index uses nine variables (benefit income, employment, household income, communication, transport, support, qualifications, living space, and home ownership) from the census to place small area blocks on a deprivation scale from 1 to 10; 10 represents the most deprived 10% of New Zealand areas, while 1 represents the least deprived 10% of areas. For analyses, deprivation was categorised into five groups: deciles 1–2 being the least deprived, and deciles 9–10 the most deprived. BMI was also categorised into three groups: less than 25 kg/m^2^, 25–30 kg/m^2^ (overweight), and 30 kg/m^2^ or higher (obese).

Women were classified as premenopausal if they had had a menstrual period in the last three months, or if their periods had stopped due to pregnancy/lactation, or use of hormonal birth control. Women were classified as postmenopausal if they reported natural menopause, surgical menopause involving bilaterial oophorectomy, or use of hormone replacement therapy (HRT). Women who did not fall into these categories, who reported surgical menopause without bilaterial oophoretoomy, and other or unknown reasons for menses cessation were classified in an ‘other amenorrhea’ category; we then assumed that those aged less than 49 years were premenopausal and those aged 49 years or more were postmenopausal based on data from New Zealand and the UK, which indicate 49 years as the median age at menopause for similarly aged birth cohorts [29], [30].

### Statistical analysis

Descriptive analyses were initially conducted to explore the variable values and summarise the data, and chi-squared tests were used to compare exposure distributions between cases and controls. Unconditional logistic regression models were used to estimate odds ratios (OR) and 95% confidence intervals (CI) for the associations between breast cancer and smoking at different points across the lifecourse for each ethnic group, adjusted for age and menopause status, and additionally adjusted by all other covariates (age at menarche, BMI, exercise, HRT, OC, maternal breast cancer, parity, alcohol, and SEP). Interactions by ethnic group were assessed using likelihood ratio tests.

Subgroup analysis and likelihood ratio tests were performed by menopausal and BMI statuses, which found no evidence of interactions (data not shown). As never smokers are different in many ways to smokers, using non-smokers as a reference group could result in confounding due to these unmeasured factors. Therefore, a range of sensitivity analyses were conducted, in which other smokers or ex-smokers were used as a reference group. For example, smokers who had stopped for less than 10 years were compared to smokers who had stopped for 10 or more years (reference group). In general, this made little difference to the results, and no difference to the interpretation of our data (data not shown), suggesting that the possibility of confounding due to these unmeasured factors was unlikely. Further sensitivity analysis was performed to investigate non-response bias, using post-stratification weights. A weight was calculated for each stratum of ethnicity*deprivation, by dividing the expected deprivation distribution of each ethnic group by the observed deprivation distribution in the controls from our study. The expected distributions were estimated from the 2002/03 New Zealand Health Survey (unpublished data), and were: 2%, 3%, 10%, 20% and 65% for Māori and Pacific women in quintiles 1 to 5 of the NZDep2006 categories, and 23%, 20%, 20%, 20% and 17% for non- Māori/non-Pacific women. Logistic regression models were then weighted using the “svy: logistic” command.

All analyses were performed using Stata version 11.2.

## Results

There were 1799 cases included in the study: 302 Māori, 70 Pacific and 1427 non- Māori/non-Pacific. Three controls were excluded due to missing age information, leaving a total of 2540 controls: 746 Māori, 191 Pacific, and 1603 non- Māori/non-Pacific. Table 1 shows the distribution of smoking behaviours by ethnic group in both cases and controls. Among the controls, higher proportions of Māori had ever smoked, and or were currently smoking, compared with the other ethnic groups. Non-Māori/non-Pacific had the lowest proportion of current smokers who smoked for less than 20 years, suggesting they have fewer new smokers starting than the other ethnic groups. Māori reported the highest proportion of young smokers, with 62% of ever smokers taking up the habit before 18 years of age. Non-Māori/non-Pacific had the highest proportion of ex-smokers who quit the habit at least 10 years prior. Pacific women reported smoking lower amounts, with 72% of current smokers having less than 10 cigarettes per day. Pacific women also had the lowest proportion of smokers who had started before the birth of their first child. Non-Māori/non-Pacific reported the highest proportion of smokers who smoked at least 5 years prior to their first birth. Māori women reported the highest prevalence of smoking during pregnancy compared to the other ethnic groups.

**Table 1:** Distribution of smoking behaviours by ethnic group.

	cases n = 1799							controls n = 2540						
	MĀORI n = 302		PACIFIC n = 70		NON-MĀORI/NON-PACIFIC n = 1427			MĀORI n = 746		PACIFIC n = 191		NON-MĀORI/NON-PACIFIC n = 1603		
Status	n	%	n	%	n	%		n	%	n	%	n	%	
Never smoked	56	18.5	29	41.4	771	54.0		209	28.0	103	53.9	869	54.2	
Ever smoked	246	81.5	40	57.1	656	46.0		535	71.7	86	45.0	731	45.6	
Current smoker	109	36.1	15	21.4	141	9.9		204	27.4	36	18.9	187	11.7	
Exsmoker	137	45.4	25	35.7	515	36.1	<0.001	331	44.4	50	26.2	544	33.9	<0.001
missing			1	1.4				2	0.3	2	1.1	3	0.2	
**Duration**														
Exsmoker <20 yrs	53	38.7	13	52.0	236	45.8		181	54.7	27	54.0	300	55.2	
Exsmoker 20+ yrs	70	51.1	11	44.0	224	43.5	0.409	118	35.7	15	30.0	195	35.9	0.593
missing	14	10.2	1	4.0	55	10.7		32	9.7	8	16.0	49	9.0	
Current <20 yrs	6	5.5	3	20.0	6	4.3		24	11.8	9	25.0	8	4.3	
Current 20+ yrs	102	93.6	12	80.0	134	95.0	0.169	178	87.3	27	75.0	179	95.7	0.001
missing	1	0.9			1	0.7		2	1					
**Initiation**														
Ever <18 yrs old	140	56.9	13	31.7	293	44.7		332	61.8	28	31.8	332	45.2	
Ever 18+ yrs old	103	41.9	27	65.9	342	52.1	0.002	188	35.0	55	62.5	385	52.5	<0.001
missing	3	1.2	1	2.4	21	3.2		17	3.2	5	5.7	17	2.3	
**Cessation**														
Stopped <10 yrs	50	36.5	11	44.0	123	23.9		103	31.1	22	44.0	91	16.7	
Stopped 10+ yrs	77	56.2	13	52.0	361	70.1	0.009	215	65.0	21	42.0	422	77.6	<0.001
missing	10	7.3	1	4.0	31	6.0		13	3.9	7	14.0	31	5.7	
**Amount**														
Exsmoker <10/day	67	48.9	13	52.0	269	52.2		167	50.5	31	62.0	292	53.7	
Exsmoker 10+/day	69	50.4	12	48.0	234	45.4	0.600	164	49.6	16	32.0	245	45.0	0.001
missing	1	0.7			12	2.3				3	6.0	7	1.3	
Current <10/day	50	45.9	9	60.0	58	41.1		86	42.2	26	72.2	64	34.2	
Current 10+/day	58	53.2	6	40.0	83	58.9	0.450	118	57.8	10	27.8	123	65.8	<0.001
missing	1	0.9												
At 20 yrs <10/day	73	36.9	9	40.9	249	50.0		173	40.2	28	45.2	299	52.5	
At 20 yrs 10+/day	123	62.1	9	40.9	231	46.4	<0.001	243	56.5	30	48.4	257	45.1	0.002
missing	2	1.0	4	18.2	18	3.6		14	3.3	4	6.5	14	2.5	
At 40 yrs <10/day	49	29.0	10	41.7	124	34.4		84	30.8	25	52.1	111	31.4	
At 40 yrs 10+/day	114	67.5	10	41.7	221	61.4	0.015	179	65.6	19	39.6	234	66.1	0.004
missing	6	3.6	4	16.7	15	4.2		10	3.7	4	8.3	9	2.54	
**Childbirth**														
Initiation before first birth	177	80.5	25	73.5	478	81.3		393	78.6	51	67.1	556	81.5	
Initiation after first birth	34	15.5	6	17.7	70	11.9	0.323	68	13.6	16	21.1	84	12.3	0.052
missing	9	4.1	3	8.8	40	6.8		39	7.8	9	11.8	42	6.2	
Duration <5 yrs before first birth	75	40.3	11	39.3	149	28.8		165	38.2	28	46.7	178	29.8	
Duration 5+ yrs before first birth	102	54.8	14	50.0	329	63.5	0.032	228	52.8	23	38.3	378	63.2	<0.001
missing	9	4.8	3	10.7	40	7.7		39	9.0	9	15.0	42	7.0	
Not during pregnancy	103	46.8	17	50.0	342	58.2		251	50.2	51	67.1	393	57.6	
Smoked during pregnancy	108	49.1	13	38.2	223	37.9	0.009	232	46.4	23	30.3	264	38.7	0.020
missing	9	4.1	4	11.8	23	3.9		17	3.4	2	2.6	25	3.7	

Status includes all study subjects while other subsections include only current and/or ex-smokers.

Table 2 presents the distribution of breast cancer risk factors among the population controls by smoking status (never/ever) for each ethnic group. Ever smokers were younger, less likely to abstain from alcohol, and more frequently from the most deprived areas than never smokers, across all the ethnic groups. Māori smokers were less likely to have used HRT, while non-Māori/non-Pacific smokers were more likely than their never smoking counterparts.

**Table 2:** Characteristics of population controls according to never/ever smoking status.

	MĀORI		PACIFIC		NON-MĀORI/NON-PACIFIC	
	Never	Ever	Never	Ever	Never	Ever
	(n = 209)	(n = 535)	(n = 103)	(n = 86)	(n = 869)	(n = 731)
**Menopausal status**						
Premenopausal	44.0	44.7	42.7	58.1	25.9	22.7
Postmenopausal	56.0	55.3	57.3	41.9	74.1	77.3
**BMI**						
<25	34.9	30.5	4.9	11.6	50.6	48.6
25–30	27.3	26.9	26.2	16.3	28.4	27.0
30+	33.5	37.9	60.2	64.0	19.0	21.9
missing	4.3	4.7	8.7	8.1	2.0	2.6
**Exercise**						
1 (least active)	27.8	30.3	17.5	23.3	23.5	21.3
2	22.5	27.3	27.2	22.1	27.1	28.2
3	21.1	16.5	17.5	17.4	22.0	23.9
4 (most active)	25.8	22.2	29.1	33.7	25.4	25.1
missing	2.9	3.7	8.7	3.5	1.9	1.5
**OC use**						
Never	18.7	20.0	67.0	47.7	20.9	18.5
Ever	79.9	79.4	31.1	52.3	78.8	81.4
missing	1.4	0.6	1.9	–	0.2	0.1
**HRT use**						
Never	83.3	88.0	98.1	96.5	76.9	71.7
Ever	15.8	10.8	1.0	3.5	22.3	27.8
missing	1.0	1.1	1.0	–	0.8	1.1
**Maternal breast cancer**						
No	93.3	89.9	90.3	91.9	92.6	91.4
Yes	3.4	5.6	2.9	1.2	6.2	6.4
missing	3.4	4.5	6.8	7.0	1.2	2.2
**Deprivation**						
1 (least deprived)	15.8	8.8	6.8	2.3	26.8	25.4
2	24.4	12.5	10.7	8.1	26.1	22.9
3	16.8	18.1	10.7	11.6	21.4	20.1
4	22	24.9	18.5	25.6	15.7	20.8
5 (most deprived)	21.1	34.7	51.5	52.3	9.8	10.8
missing	–	–	1.9	–	0.2	–
**Frequency of alcohol**						
non drinker	23.4	18.5	69.9	39.5	16.2	11.8
monthly	25.4	35.1	21.4	32.6	23.6	18.6
2–4/month	21.1	18.1	5.8	14.0	21.2	17.5
2–3/week	22.5	16.1	2.9	4.7	19.2	19.8
4+/week	7.7	11.4	–	9.3	19.8	32.3
missing	–	0.8	–	–	–	–
**Mean (SD) age**	52.7 (11.5)	51.1 (10.5)	52.4 (12.4)	47.9 (11.1)	59.4 (12.1)	59.2 (11.7)
**Mean (SD) age at menarche**	12.8 (1.6)	12.6 (1.7)	13.4 (1.8)	13.2 (1.9)	12.9 (1.5)	12.9 (1.4)
**Mean (SD) age at first birth**	24.3 (5.2)	22.2 (4.7)	24.3 (5.0)	23.2 (4.4)	25.9 (4.8)	24.7 (5.1)
**Mean (SD) live births**	2.3 (1.3)	2.5 (1.3)	2.7 (1.5)	2.7 (1.5)	2.4 (1.2)	2.4 (1.2)

The associations between smoking status and duration, and breast cancer are presented in Table 3 by ethnic group. Māori and Pacific women show similar patterns, with all smoking categories showing increased odds of breast cancer compared to never smokers; these associations persisted after adjustment for all covariates, although in some cases the increased risks were greatly reduced. There was no clear association between smoking status and breast cancer for non-Māori/non-Pacific women. Non-Māori/non-Pacific ex-smokers had statistically significant increased odds of breast cancer when smoking duration was 20 years or more, and this remained significant in the fully adjusted model (OR 1.31, 95% CI 1.03 to 1.66). Māori showed increased odds of breast cancer with increasing duration among current smokers (<20 years OR 1.61, 95% CI 0.55 to 4.74; 20+ years OR 2.03, 95% CI 1.29 to 3.22). While there was evidence of a detrimental effect of longer smoking duration, there was no evidence that smoking before 18 years of age increased the likelihood of breast cancer more than starting later in life. There was a clear pattern of shorter duration since smoking cessation being associated with increased likelihood of breast cancer, and this was apparent for all ethnic groups.

**Table 3:** Associations between smoking status and duration, and breast cancer incidence by ethnic group.

STATUS & DURATION	MĀORI		PACIFIC		NON-MĀORI/NON-PACIFIC		
	age & menopause adjusted	covariate adjusted*	age & menopause adjusted	covariate adjusted*	age & menopause adjusted	covariate adjusted*	P (ethnic interaction)
Never smoked	1 (Reference)	1 (Reference)	1 (Reference)	1 (Reference)	1 (Reference)	1 (Reference)	
Ever smoked	1.82 (1.30 to 2.54)	1.49 (1.03 to 2.15)	1.69 (0.95 to 2.98)	1.23 (0.59 to 2.58)	1.02 (0.89 to 1.18)	1.02 (0.87 to 1.19)	0.147
Current smoker	2.30 (1.56 to 3.39)	1.90 (1.24 to 2.92)	1.79 (0.95 to 3.39)	1.25 (0.44 to3.52)	0.85 (0.67 to 1.08)	0.76 (0.58 to 1.00)	
Exsmoker	1.57 (1.09 to 2.25)	1.28 (0.86 to 1.91)	1.53 (0.72 to 3.23)	1.22 (0.54 to 2.76)	1.08 (0.93 to 1.27)	1.11 (0.93 to 1.31)	0.026
**Duration**							
Ex <20 yrs	1.20 (0.78 to 1.85)	1.00 (0.62 to 1.62)	1.72 (0.78 to 3.80)	1.18 (0.33 to 4.20)	0.87 (0.72 to 1.06)	0.97 (0.78 to 1.20)	
Ex 20+ yrs	2.07 (1.35 to 3.15)	1.59 (0.97 to 2.59)	2.53 (1.04 to 6.15)	1.85 (0.54 to6.28)	1.37 (1.10 to 1.70)	1.31 (1.03 to 1.66)	0.426
Current <20 yrs	1.65 (0.60 to 4.51)	1.61 (0.55 to 4.74)	1.19 (0.28 to 5.05)	2.80 (0.39 to 20.07)	0.77 (0.26 to 2.25)	0.57 (0.17 to 1.88)	
Current 20+ yrs	2.39 (1.61 to 3.56)	2.03 (1.29 to 3.22)	1.53 (0.68 to 3.45)	0.88 (0.27 to 2.87)	0.86 (0.67 to 1.10)	0.79 (0.60 to 1.04)	0.023
**Initiation**							
Ever <18 yrs old	1.80 (1.25 to 2.59)	1.43 (0.96 to 2.13)	1.79 (0.80 to 3.99)	0.98 (0.32 to 3.05)	0.99 (0.82 to 1.19)	0.95 (0.77 to 1.16)	
Ever 18+ yrs old	1.97 (1.34 to 2.89)	1.68 (1.10 to 2.57)	1.72 (0.92 to 3.22)	1.40 (0.63 to 3.09)	1.02 (0.86 to 1.23)	1.05 (0.87 to 1.28)	0.198
**Cessation**							
Stopped <10 yrs	2.63 (1.69 to 4.10)	2.09 (1.27 to 3.46)	2.13 (0.95 to 4.80)	1.98 (0.64 to 6.14)	1.70 (1.28 to 2.26)	1.55 (1.14 to 2.09)	
Stopped 10+ yrs	1.28 (0.86 to 1.90)	1.02 (0.65 to 1.60)	2.17 (0.97 to 4.86)	1.28 (0.36 to 4.56)	0.98 (0.83 to 1.17)	1.06 (0.88 to 1.28)	0.953

* adjusted for age, menopause status, age at menarche, BMI, exercise, HRT, OC, maternal breast cancer, parity, alcohol, and SEP.

The associations between the quantity of cigarettes consumed per day and breast cancer are presented in Table 4 by ethnic group. Overall there was little evidence that smoking 10 or more cigarettes per day increased the likelihood of breast cancer more than smoking less than 10 per day in any ethnic group, or smoking category. Adjusted associations between amount per day and breast cancer were rerun using the higher cut point of 20 or more cigarettes per day and only significant results were found among Māori smokers at ages 20 years (<20/day OR 1.51, 95% CI 1.02 to 2.25; 20+/day OR 1.87, 95% CI 1.12 to 3.13) and 40 years (<20/day OR 1.73, 95% CI 1.13 to 2.64; 20+/day OR 2.69, 95% CI 1.59 to 4.54).

**Table 4:** Associations between smoking amount and breast cancer incidence by ethnic group.

STATUS & INTENSITY	MĀORI		PACIFIC		NON-MĀORI/NON-PACIFIC		
	age & menopause adjusted	covariate adjusted*	age & menopause adjusted	covariate adjusted*	age & menopause adjusted	covariate adjusted*	P (ethnic interaction)
Never	1 (Reference)	1 (Reference)	1 (Reference)	1 (Reference)	1 (Reference)	1 (Reference)	
Exsmoker <10/day	1.49 (0.99 to 2.26)	1.29 (0.81 to 2.05)	1.47 (0.68 to 3.19)	1.12 (0.37 to 3.36)	1.05 (0.86 to 1.27)	1.17 (0.95 to 1.44)	
Exsmoker 10+/day	1.59 (1.05 to 2.41)	1.18 (0.74 to 1.89)	2.67 (1.13 to 6.29)	2.39 (0.67 to 8.54)	1.10 (0.90 to 1.35)	1.05 (0.84 to 1.31)	0.795
Current <10/day	2.64 (1.64 to 4.27)	2.36 (1.37 to 4.08)	1.19 (0.49 to 2.91)	1.04 (0.30 to 3.56)	1.02 (0.70 to 1.48)	0.97 (0.64 to 1.46)	
Current 10+/day	2.12 (1.35 to 3.33)	1.76 (1.05 to 2.94)	2.16 (0.71 to 6.62)	1.31 (0.20 to 8.63)	0.78 (0.58 to 1.06)	0.69 (0.50 to 0.96)	0.049
At 20 yrs old <10/day	1.72 (1.14 to 2.60)	1.47 (0.94 to 2.32)	1.14 (0.47 to 2.76)	1.12 (0.36 to 3.52)	0.95 (0.78 to 1.16)	1.02 (0.82 to 1.26)	
At 20 yrs old 10+/day	2.14 (1.47 to 3.12)	1.67 (1.11 to 2.52)	1.06 (0.45 to 2.52)	0.42 (0.13 to 1.43)	1.02 (0.83 to 1.25)	0.95 (0.76 to 1.18)	0.154
At 40 yrs old <10/day	2.18 (1.37 to 3.46)	1.94 (1.16 to 3.23)	1.34 (0.57 to 3.14)	1.41 (0.50 to 4.02)	1.29 (0.98 to 1.70)	1.42 (1.05 to 1.92)	
At 40 yrs old 10+/day	2.33 (1.59 to 3.42)	1.98 (1.29 to 3.04)	1.91 (0.80 to 4.59)	1.48 (0.44 to 5.02)	1.11 (0.90 to 1.37)	0.98 (0.78 to 1.24)	0.022

* adjusted for age, menopause status, age at menarche, BMI, exercise, HRT, OC, maternal breast cancer, parity, alcohol, and SEP.

The associations between smoking before and during pregnancy, and breast cancer are presented by ethnic group in Table 5. There was some evidence to suggest smoking prior to first birth increased the likelihood of breast cancer for Māori women. There was also a pattern of increased odds of breast cancer with increasing smoking duration prior to first birth in Māori and Pacific women, which remained in the fully adjusted model. Overall, there was little evidence of increased likelihood of breast cancer among smokers who smoked during a pregnancy.

**Table 5:** Associations between smoking before first birth and breast cancer incidence by ethnic group.

INITIATION & DURATION	MĀORI		PACIFIC		NON-MĀORI/NON-PACIFIC		
	age & menopause adjusted	covariate adjusted*	age & menopause adjusted	covariate adjusted*	age & menopause adjusted	covariate adjusted*	P (ethnic interaction)
Never	1 (Reference)	1 (Reference)	1 (Reference)	1 (Reference)	1 (Reference)	1 (Reference)	
Initiation before first birth	1.83 (1.29 to 2.60)	1.49 (1.00 to 2.23)	1.67 (0.88 to 3.18)	1.25 (0.48 to 3.26)	0.98 (0.84 to 1.14)	1.01 (0.85 to 1.20)	
Initiation after first birth	1.70 (1.02 to 2.84)	1.34 (0.76 to 2.38)	1.34 (0.48 to 3.76)	1.69 (0.40 to 7.15)	0.98 (0.70 to 1.37)	0.91 (0.63 to 1.32)	0.235
Duration <5 yrs before first birth	1.70 (1.13 to 2.56)	1.24 (0.78 to 1.99)	1.37 (0.60 to 3.12)	1.15 (0.34 to 3.91)	0.98 (0.77 to 1.24)	0.89 (0.69 to 1.16)	
Duration 5+ yrs before first birth	1.89 (1.29 to 2.79)	1.68 (1.08 to 2.63)	1.99 (0.90 to 4.43)	1.70 (0.52 to 5.56)	0.98 (0.82 to 1.17)	1.06 (0.87 to 1.30)	0.274
Not during pregnancy	1.59 (1.09 to 2.33)	1.38 (0.90 to 2.12)	1.16 (0.58 to 2.32)	1.04 (0.40 to 2.70)	0.98 (0.83 to 1.17)	1.06 (0.88 to 1.29)	
During pregnancy	1.82 (1.25 to 2.66)	1.39 (0.90 to 2.16)	1.95 (0.87 to 4.38)	1.60 (0.46 to 5.53)	0.98 (0.80 to 1.20)	0.91 (0.73 to 1.14)	0.152

* adjusted for age, menopause status, age at menarche, BMI, exercise, HRT, OC, maternal breast cancer, parity, alcohol, and SEP.

Overall, the effect of weighting the controls for differential non-response attenuated the effect estimates slightly for Māori (e.g. ever smoked OR changed from 1.49 to 1.43 (95% CI 0.95 to 2.17)); for Pacific, the estimates, although unstable, showed some evidence of a strengthening of effect (e.g. ever smoked OR changed from 1.23 to 1.36 (95% CI 0.49 to 3.76)); and for non-Māori/non-Pacific, there was almost no change in effect (e.g. ever smoked OR remained at 1.02 (95% CI 0.86 to 1.21)).

To explore the relative confounding effect of variables across ethnic groups, each potential confounder was added individually to models which included ‘ever smoking’ and we checked whether the OR for ever smoking and breast cancer then changed (Table 6). Among Māori women, adding potential confounders only slightly affected the OR estimate for ever smoking. The greatest attenuation in OR was after adjustment for maternal breast cancer, followed by BMI adjustment. Among Pacific women, the greatest attenuation was after adjustment for exercise, whereas among non-Māori/non-Pacific women, no single variable had a strong confounding effect.

**Table 6:** The effect of adjusting associations between smoking and breast cancer for potential confounders individually.

COVARIATE	MĀORI	PACIFIC	NON-MĀORI/NON-PACIFIC
Never	1 (Reference)	1 (Reference)	1 (Reference)
base model*	1.65 (1.17 to 2.33)	1.60 (0.90 to 2.85)	0.99 (0.86 to 1.15)
age at menarche	1.65 (1.17 to 2.34)	1.56 (0.87 to 2.78)	1.01 (0.87 to 1.17)
BMI	1.59 (1.13 to 2.25)	1.51 (0.83 to 2.77)	0.99 (0.85 to 1.15)
exercise	1.62 (1.15 to 2.30)	1.23 (0.67 to 2.26)	0.97 (0.83 to 1.12)
OC	1.60 (1.13 to 2.26)	1.51 (0.84 to 2.71)	1.00 (0.87 to 1.16)
maternal BC	1.58 (1.12 to 2.24)	1.59 (0.89 to 2.85)	1.00 (0.86 to 1.16)
alcohol	1.73 (1.22 to 2.44)	1.67 (0.91 to 3.08)	1.01 (0.87 to 1.17)
HRT	1.64 (1.17 to 2.32)	1.57 (0.88 to 2.80)	1.01 (0.87 to 1.16)
parity	1.66 (1.17 to 2.34)	1.67 (0.91 to 3.07)	0.99 (0.85 to 1.14)

* ever smoked status, adjusted for age, menopause status, age at menarche, SEP.

## Discussion

This study has found little evidence of associations between cigarette smoking and breast cancer incidence in non-Māori/non-Pacific women, but increased risks were observed for Māori and Pacific smokers. In the latter ethnic groups, elevated risks were observed for both ex-smokers and current smokers. Length of time since quitting smoking was inversely associated with the risk of breast cancer, and elevated risks were observed for those who smoked for 20 years of more. The findings in Pacific women were in general not statistically significant, but the numbers were relatively small, and the odds ratios were consistent with those found for Māori women. Furthermore, variations were found in the magnitude of the change in the smoking effects after adjustment for confounders among the different ethnic groups.

Findings of the current study are consistent with several previous studies which found a strong association between long duration of smoking and breast cancer [8], [12], [13], [31]. We observed increased risks in current Māori smokers and non-Māori/non-Pacific women who are ex-smokers, and duration of smoking before first birth was also associated with an elevated risk of breast cancer. This is consistent with several previous studies that have observed elevated risk among women who smoked more than five years prior to first birth [8], [9], [10], [12], [14], [31], [32], [33]. Two meta-analyses [34], [35] concluded that there was no association between smoking prior to first birth and breast cancer; however, these studies did not assess the length of smoking duration prior to birth (summary relative risks ranged from 7% to 10% greater risk among those who smoked prior to first pregnancy compared with those who never smoked).

A large collaborative study found that smoking appears to have little or no independent effect on breast cancer risk beyond the risk conferred by drinking alcohol [36]. The only exposure variable examined by the study however, was ever smoking, and a 3% increased risk for ever smoking compared to never smoking was observed in the reanalysis of data from 53 epidemiological studies.

Most studies that have examined associations between smoking and breast cancer for premenopausal and postmenopausal women separately, have observed no meaningful difference in risk by menopausal status [6]. Similarly, estimates from the current study did not materially change when stratified by menopausal status, and likelihood ratio tests produced no evidence of interaction between smoking and menopausal status among any ethnic group. Nor was there any evidence of interaction found between BMI and smoking among any ethnic group.

Among non-Māori/non-Pacific, statistically significant elevated risks were only observed among ex-smokers who smoked for more than 20 years, and smokers who had stopped for less than 10 years. Our findings regarding the length of time since stopping smoking are consistent with some previous studies [13], [22], [37], and in contrast to others which found no association between time since smoking cessation and breast cancer risk [8], [10], [14], [38]. It has been posited that although smoking may malignantly alter breast cells at an early age, continuing to smoke could slow proliferation [32]. Thereby a longer latency could be evident in current smokers [39] and explain some of the observed elevated risks in ex-smokers compared with current smokers [9], [10], [38].

The association between smoking and breast cancer was stronger for Māori than in the other ethnic groups. Māori women have extremely high rates of smoking and the different effects observed for different ethnic groupings could be affected by these. Few populations have such high smoking rates among women; in 2009 the smoking prevalence for Māori women was more than three times that for non-Māori: 48.3% compared with 16.2% respectively [40]. The ethnic differences observed could be an artefact of differential recall bias among non-Māori/non-Pacific, whereby they are more conscious of the harms associated with smoking, and it is therefore less socially acceptable to smoke, and report smoking.

The current study had several limitations, including the potential selection and recall biases commonly associated with case-control studies involving patient interviews. We know from comparison of control distributions with distributions of the New Zealand Deprivation Index that affluent women are over-represented and deprived women are under-represented in the study. Therefore, control weighting adjustment was applied to account for this difference, and this did not materially change our results. The response rates were particularly poor among Pacific controls (15%) and therefore this group is unlikely to be representative of the population of Pacific women in New Zealand. However, there is a paucity of research in this population group and more is needed to increase the limited evidence available regarding breast cancer risk for Pacific women. There were small numbers in some strata, especially among Pacific women, which limit the precision of the effect estimates. Limited numbers also restricted the analyses conducted with regard to categorical comparisons and ability to assess differences among breast cancer subtypes.

Another limitation of this study is the potential for residual confounding. For example, the odds ratio for ever smokers in Māori fell from 1.82 to 1.49 when the analyses were adjusted for BMI, exercise, HRT, OC, maternal breast cancer, parity, alcohol and SEP; the odds ratio in Pacific women fell from 1.69 to 1.23. Thus, about 40% of the excess risk in Māori, and more than half of the excess risk in Pacific women was removed by adjustment for these confounders. Given that we did not have perfect confounder information (as with all studies of this type), it is likely that the excess risk would have reduced even further if we had been able to obtain ‘perfect’ confounder information. Thus, if one considered only the findings for ever smokers (current smokers, and ex-smokers) it is possible that the observed excess risk could be due to residual confounding. However, when we considered more detailed smoking information, including smoking duration, the associations were stronger and the reduction in excess risk was relatively less, when adjusting for potential confounders. Thus, it appears to be unlikely that all of the excess risks we observed were due to residual confounding.

Adjustment for confounding factors affected the smoking odds ratios differently among the ethnic groups. The magnitude of the change following the adjustment, and the relative importance of the relationship between smoking and factors such as BMI, exercise, OC, and family history of breast cancer, are likely to differ between ethnic groups and populations. The amount of residual confounding is also likely to vary and affect the findings for specific populations depending on the suitability of adjustment for each population. Therefore, the appropriateness of adjustments for potential confounders could play a role in the observed inconsistent findings for smoking and breast cancer in various populations [6], [8], [13], [15], [36].

Previous studies have found that positive associations between active smoking and breast cancer were strengthened when passive smokers were excluded from the reference comparison [12], [41], [42]. We were not able to examine the effect of passive smoking in this study. If high exposure to passive smoking was more frequent among control women, this could have biased our estimates of smoking and breast cancer towards the null. Furthermore, duration of both smoking and passive smoking is also important [13], [42]; if exposure to passive smoke over an extended duration increased breast cancer risk more than smoking for a more limited period, this could produce spurious results for smokers exposed to long-term environmental tobacco smoke.

In conclusion, there was no clear pattern for cigarette smoking and breast cancer incidence in non-Māori/non-Pacific women, but clear patterns of higher risk were observed for Māori and Pacific smokers. These findings suggest that smoking could play a role in the ethnic disparities which have been observed in the distribution of breast cancer in New Zealand. Lowering the prevalence of cigarette smoking, especially among Māori and Pacific women, could be important for reducing breast cancer incidence.
